# Association of stigma with mental health and quality of life among Indonesian COVID-19 survivors

**DOI:** 10.1371/journal.pone.0264218

**Published:** 2022-02-23

**Authors:** Joni Wahyuhadi, Ferry Efendi, Makhyan Jibril Al Farabi, Iman Harymawan, Atika Dian Ariana, Hidayat Arifin, Qorinah Estiningtyas Sakilah Adnani, Inbar Levkovich

**Affiliations:** 1 Department of Neurosurgery, Soetomo General Hospital, Faculty of Medicine, Universitas Airlangga, Surabaya, Indonesia; 2 Faculty of Nursing, Universitas Airlangga, Surabaya, Indonesia; 3 Department of Cardiology and Vascular Medicine, Universitas Airlangga, Surabaya Indonesia; 4 Faculty of Economy and Business, Universitas Airlangga, Surabaya, Indonesia; 5 Faculty of Psychology, Universitas Airlangga, Surabaya, Indonesia; 6 Department of Medical-Surgical, Critical, Emergency, and Disaster Nursing, Faculty of Nursing, Universitas Padjadjaran, Bandung, Indonesia; 7 Department of Public Health, Faculty of Medicine, Universitas Padjadjaran, Bandung, Indonesia; 8 Faculty of Graduate Studies, Oranim Academic College of Education, Kiryat Tiyon, Israel; Haramaya University, ETHIOPIA

## Abstract

**Background and objective:**

Coronavirus disease 2019 (COVID-19) survivors face societal stigma. The study aims to analyze the association of this stigma with the mental health and quality of life of COVID-19 survivors.

**Methods:**

In this cross-sectional study, we observed 547 adults who were previously documented as severe acute respiratory syndrome coronavirus (SARS-CoV-2) positive by a polymerase chain reaction (PCR) test, treated in a hospital or an emergency hospital and proven to be SARS-CoV-2 negative by their latest PCR test. We adopted the Berger HIV Stigma Scale to measure stigma; the World Health Organization Quality of Life Brief Form to measure quality of life; and the Mental Health Inventory-38 to measure mental health. The chi-square and binary logistic regression tests were used to find the correlation between the variables.

**Results:**

The multivariate analysis revealed that medium stigma was more likely related to quality of life and mental health than low stigma. Females were less likely to experience stigma related to mental health than men, and respondents who worked as laborers and entrepreneurs were less likely to experience stigma related to mental health than those who worked as civil workers/army personnel/teachers/lecturers. COVID-19 survivors experienced medium stigma in society and lower quality of life and mental health status. We found that quality of life and mental health were affected by stigma, sex, and occupation.

**Conclusion:**

COVID-19 survivors are a vulnerable group that is most at risk when they return to their communities. Creating a safe environment and providing respectful care, including addressing complex stigma factors, is vital for developing appropriate interventions.

## Introduction

The coronavirus disease 2019 (COVID-19) pandemic has affected the worldwide population, thus creating a burden of disease and mortality and an unprecedented impact on social life [1, 2]. According to the World Health Organization (WHO), on February 18, 2021, more than 109 million cases had been confirmed and approximately two million deaths had occurred across 223 countries [3]. Insufficient knowledge and contradictory information about the severity of and protection against severe acute respiratory syndrome coronavirus (SARS-CoV-2) has increased anxiety among the population [1]. This uncertainty and anxiety have led people to easily believe vague and biased information from the media, social media, and self-proclaimed experts [4]. At the same time, this rapidly spreading and unpredictable pandemic has led to the imposition of social stigma and discrimination against COVID-19 survivors [5, 6]. According to the WHO, “all efforts must be taken to scientifically destigmatize COVID-19 instead of statutory sermons by lawmakers” [7]. Usually, stigma develops when people are afraid and believe that COVID-19 survivors are still contagious.

Indonesia is one of the countries that has suffered remarkably in terms of the number of COVID-19 cases. With approximately 270 million inhabitants of over 300 ethnicities scattered across 34 provinces, morbidity and mortality associated with COVID-19 in Indonesia are among the highest the world wide, and together with India and Sri Lanka, Indonesia continues to report the highest number of new cases and new deaths in Southeast Asia, thus contributing to the global burden of COVID-19 cases [8]. Global statistics on February 17, 2021 revealed that Indonesia reported a large number of new cases (9687) and 192 new deaths, which marked more than a million confirmed cases and 33788 deaths since the first case was reported in March 2020 [9].

Although the Indonesian government has implemented certain interventions, including quarantine, travel restrictions, social distancing, and health education (e.g., encouraging wearing masks and hand washing), the virus continues to spread through community transmission [10]. The fundamental strategy for hospitals and health workers has been for essential core health services to be intensified to deal with COVID-19 cases; however, the country has struggled to prevent community transmission. The high number of health worker deaths due to COVID-19 invited speculation on health workers’ job insecurity, insufficient health supplies, and inadequate health facilities and resources (10). Thus, the trend of a high number of newly confirmed cases and new deaths due to COVID-19 is still tracking nationally [11].

As Indonesia continues its effects to reduce the number of new confirmed cases and new deaths, the incidence of stigma toward COVID-19 survivors in social media has become a serious concern [11]. People who have recovered from SARS-CoV-2 infection may experience multiple types of stigma, such as anticipated stigma, i.e., fear of being tested for SARS-CoV-2, perceived stigma, i.e., feeling judged by others, and internalized stigma, i.e., experiencing shame and self-rejection [1]. Many COVID-19 survivors have even reported discrimination, stereotyping, and job loss as a result of people associating them with a deadly disease [6, 12]. Social stigma has also negatively affected social justice for COVID-19 survivors because a stigmatized person cannot actively participate in society [13]. Some COVID-19 survivors have suffered severe mental distress even after discharge and rehabilitation [14, 15]. All of these phenomena can reduce the quality of life and mental health of COVID-19 survivors [16, 17]

The Quality of Life (QoL) scale is an important measurement of the impact of COVID-19 infection on the physical, mental, and social domains of COVID-19 survivors. Assessing QoL helps health care providers identify key factors affecting QoL and recognize the aspects of COVID-19 management that can be improved to enhance the QoL of COVID-19 survivors [18]. A total of more than one million confirmed recovered COVID-19 cases out of more than six million tested in Indonesia raises the question of the needs of COVID-19 survivors when discharged from the hospital [19]. An understanding of COVID-19 survivors’ stigma associated with mental health and QoL is critical and will enable policy-makers to better understand the patterns of the pandemic and design more targeted programs for this group. To date, few studies have used primary data to evaluate this stigma and its impact on COVID-19 survivors, and none have focused on Indonesia. Therefore, the present study aimed to expand upon and quantify stigma and its impact on the QoL and mental health status of the general community of COVID-19 survivors.

## Materials and methods

### Design and participants

We conducted a cross-sectional study of adult COVID-19 survivors in East Java Province, one of Indonesia’s 34 provinces. According to statistics, East Java has one of the highest numbers of confirmed COVID-19 cases, deaths, and recoveries [7, 9]. Adults aged 20 or older who recovered from COVID-19 in Indonesia were recruited from a COVID-19 Survivors Community registry. The study was conducted over two months from October 1 to December 1, 2020. The number of required samples was calculated using a 95% confidence level, and an assumed 50% distribution of results [19], with a minimum sample size of 334 required. Participants in the study were COVID-19 survivors who were previously diagnosed as SARS-CoV-2 positive from a PCR test, treated in hospital, and proven to be SARS-CoV-2 negative by their latest PCR test. The COVID-19 survivors enrolled in our study were defined as older than 20 years of age and of either gender by convenience sampling. COVID-19 survivors were eligible for the study if they were willing and able to participate and provided online informed consent. All participants provided digitally signed informed consent. A total of 547 COVID-19 survivors agreed and consented to participate in the study. This study was approved by the Health Ethics Committee, Faculty of Nursing, Airlangga University, under reference number 2105-KEPK. The Strengthening the Reporting of Observational studies in Epidemiology (STROBE) statement was used as the standard for writing this study, and all of the methods used were performed in accordance with the relevant guidelines and regulations [20].

### Procedures

Recruitment of participants was conducted through an online platform, the COVID-19 Survivors Community registry. Potential respondents were identified from this registry, and an invitation via a one-to-one private WhatsApp message was sent to each survivor. Their responses were obtained by data collectors (aged > 20 years) trained in research methods, and they were neither students nor trainees. Once the data collectors received feedback from the COVID-19 survivors, they were assessed for eligibility against certain criteria. The respondents who met the criteria were provided with brief information about the study, and those who agreed to participate were enrolled and invited to participate via questionnaires, which were circulated through an online platform. Those who were interested in joining the survey were asked to voluntarily fill out the online form.

In this study, we measured three aspects of post-COVID-19 life: stigma, QoL, and mental health. All the questionnaires were translated into Bahasa Indonesia and pilot tested prior to the study. Validation was conducted before using this measurement tool. Stigma among COVID-19 survivors was measured using the Berger HIV Stigma Scale questionnaire, which was adapted for COVID-19 stigma [21]. Cronbach’s alpha was used to measure the internal consistency of the scale, and factorial analysis was used to adapt the questionnaire. Then, the questionnaire responses were divided using the mean score into three categories: low, medium, and high.

The second questionnaire utilized was the WHO Quality of Life Brief Form (WHOQOL-BREF), which originally contained 26 items. The current study used the WHOQOL-BREF Indonesian version [22], which has proven to be reliable and valid across many different populations [23]. A five-point Likert scale was used for the WHOQOL-BREF. Each item was scored from 1 (the worst condition) to 5 (the best condition), with higher scores representing a better QoL; moreover, questions 3, 4, and 26 had a negative value. In addition, the WHOQOL-BREF includes four domains: physical (questions 3, 4, 10, 15, 16, 17, and 18), psychological (questions 5, 6, 7, 11, 19, and 26), social (20, 21, and 22), and environmental (8, 9, 12, 13, 14, 23, 24, and 25) [24]. Questions 1 and 2 ask the participants to assess their overall QoL and health in general. We categorized the total questionnaire responses into lower (< mean) and higher (> mean) QoL based on a previous study [25].

The Mental Health Inventory-38 was used to assess the mental health state of COVID-19 survivors [26]. The questionnaire consists of two dimensions: psychological well-being and psychological distress. These scales encompass various subscales: positive affect and emotional ties and anxiety, depression, and loss of behavioral/emotional control. Most items have a six-point Likert scale, while two have a 5-point scale. Each point was associated with the frequency or intensity level of the behaviors, feelings, or thoughts the person experienced. Higher scores indicated a higher level of overall mental health and its specific dimensions. Then, we categorized the total questionnaire answers into lower (< mean) and higher (> mean) mental health. Moreover, we only used the total scores and did not include the subscales, as indicated in previous studies [26, 27].

### Statistical analysis

Distributions of the characteristics of respondents were represented using descriptive statistics. The chi-square test was used to determine the association of stigma with mental health and quality of life faced by COVID-19 survivors. Binary logistic regression tests were performed by adjusting all variables with a p value < 0.05. The associations among variables were measured as odds ratios and 95% confidence intervals (CIs). The regression model met the requirements of both the omnibus (p value < 0.05) and the Hosmer & Lemeshow tests for goodness of fit (p value > 0.05). All statistical analyses were performed using SPSS version 20 (IBM, Chicago).

## Results

### Characteristics of the respondents

Table 1 depicts the characteristics of the respondents. Between October 1 and December 1, 2020, we collected responses from 580 COVID-19 survivors who voluntarily agreed to join this study. Of these, 33 individuals were excluded because they were under 20 years old; thus, the final number was 547 respondents. The baseline demographic characteristics showed a balance of male and female genders. The younger age group (52.65%) dominated over older age groups. In our study, we found that most of the respondents (287, 52.47%) did not know from whom they had contracted COVID-19 and that a majority of them isolated in the hospital (368, 67.28%). Our findings also revealed that most of the participants recovered in less than a month (452, 82.63%) and did not show any symptoms (485, 88.67%). The prevalence of medium stigma due to COVID-19 was 70.02%; the prevalence of lower QoL was 45.52%; and the prevalence of lower mental health was 49.54%. Further information about the respondents’ characteristics is available in Table 1.

**Table 1:** Characteristics of respondents (n = 547).

Variable	n	%
Sex		
Male	275	50.27
Female	272	49.73
Age		
20–30 years old	288	52.65
31–40 years old	156	28.52
>40 years old	103	18.83
Highest education level		
School	178	32.54
University	369	67.46
Occupation		
Civil/Army/Teacher/Lecturer	95	17.37
Laborer	66	12.07
Entrepreneur	113	20.66
Others	273	49.91
Salary		
Less than 1,768,776 IDR (1 USD equal to 14,000 IDR)	92	16.82
More than 1,768,777 IDR	455	83.18
Marital status		
Unmarried	208	38.03
Married	339	61.97
Pregnancy		
No	527	96.34
Yes	20	3.66
Ethnicity		
Javanese	496	90.68
Others	51	9.32
Contracted COVID-19		
Through public places	127	23.22
Through COVID-19 carrier	133	24.31
Unknown	287	52.47
Place of care		
Hospital	157	28.70
Emergency hospital	390	71.30
Place of isolation		
At home or another place	179	32.72
At hospital	368	67.28
Recovery duration		
Less than a month	452	82.63
A month or more	95	17.73
Symptoms		
No	485	88.67
Yes	62	11.33
Stigma		
Low	80	16.45
Medium	383	70.02
High	74	13.53
Quality of Life		
Lower	249	45.52
Higher	298	54.48
Mental Health		
Lower	271	49.54
Higher	276	50.46

### Bivariate analysis

Table 2 provides detailed results for the bivariate analysis and shows the variables related to QoL and mental health status. We found that the stigma faced by COVID-19 survivors (p<0.001) had a significant correlation with QoL and mental health. The sex (p = 0.002) and occupation (p = 0.038) of the COVID-19 survivors had a significant correlation with mental health.

**Table 2:** Bivariate analysis of quality of life and mental health among COVID-19 survivors in Indonesia.

Variable	Quality of Life			Mental Health		
	Lower	Higher	*p*	Lower	Higher	*p*
Sex						
Male	128	147	0.629	155	120	0.002***
Female	121	151		118	154	
Age						
20–30 years old	126	162	0.681	148	140	0.604
31–40 years old	74	82		78	78	
>40 years old	49	54		47	56	
Highest educational level						
School	87	91	0.274	86	92	0.605
University	162	207		187	182	
Occupation						
Civil/Army/Teacher/Lecturer	40	55	0.778	54	41	0.038**
Laborer	32	34		41	25	
Entrepreneur	49	64		55	58	
Others	128	145		123	150	
Salary						
Less than 1,768,776 IDR	37	55	0.263	44	48	0.661
More than 1,768,777 IDR	212	243		229	226	
Marital status						
Unmarried	92	116	0.635	107	101	0.574
Married	157	182		166	173	
Pregnancy						
No	239	288		263	264	0.993
Yes	10	10	0.682	10	10	
Ethnicity						
Java	223	273	0.411	242	254	0.103
Others	26	25		31	20	
Contracted COVID-19						
Through public places	58	69	0.640	64	63	0.978
Through COVID-19 carrier	56	77		67	66	
Unknown	135	152		142	145	
Place of care						
Hospital	77	80	0.294	78	79	0.946
Emergency hospital	172	218		195	195	
Place of isolation						
At home or another place	79	100	0.650	86	93	0.543
At hospital	170	198		187	181	
Recovery duration						
Less than a month	210	242	0.336	229	223	0.441
A month or more	39	56		44	51	
Symptoms						
No	222	263		244	241	0.600
Yes	27	35	0.787	29	33	
Stigma						
Low	22	68		25	65	
Medium	182	201	0.000***	197	186	0.000***
High	45	29		51	23	

*p<0.1

**p<0.05

***p<0.01

### Multivariate analysis

Table 3 presents the detailed results of the multivariate analysis. We omitted salary, pregnancy status, and symptoms because those factors failed to meet the threshold for significance. The results were adjusted for other potential confounders, as shown in Table 2. A high level of stigma was positively correlated with lower QoL and lower mental health status [p = 0.038; CI = 1.032–2.946; AOR = 1.744 and p = 0.038; CI = 1.032–2.946; AOR = 1.744, respectively]. Females were less likely to experience stigma related to mental health [p = 0.003; CI = 0.393–0.830; AOR = 0.571] than men. We also discovered that laborers [p = 0.047; CI = 0.351–0.992; AOR = 0.590] and entrepreneurs [p = 0.030; CI = 0.266–0.934; AOR = 0.498] were less likely to experience stigma related to mental health than respondents who worked in the civil/army/teaching/lecturing fields. More detailed results can be found in Table 3.

**Table 3:** Multivariate analysis of quality of life and mental health among COVID-19 survivors in Indonesia.

Variable	Quality of Life			Mental Health		
	AOR	CI 95%	p	AOR	CI 95%	p
Sex						
Male	Ref.			Ref.		
Female	0.963	[0.667–1.391]	0.842	0.571	[0.393–0.830]	0.003***
Age						
20–30 years old	Ref.			Ref.		
31–40 years old	1.305	[0.746–2.282]	0.351	0.682	[0.386–1.206]	0.188
>40 years old	1.107	[0.655–1.870]	0.705	0.890	[0.521–1.520]	0.670
Highest educational level						
School	Ref.			Ref.		
University	0.898	[0.598–1.348]	0.603	1.381	[0.911–2.094]	0.129
Occupation						
Civil/Army/Teacher/Lecturer	Ref.			Ref.		
Laborer	1.430	[0.857–2.387]	0.171	0.590	[0.351–0.992]	0.047**
Entrepreneur	1.216	[0.662–2.234]	0.528	0.498	[0.266–0.934]	0.030**
Others	1.222	[0.764–1.954]	0.403	0.788	[0.491–1.264]	0.322
Marital status						
Unmarried	Ref.			Ref.		
Married	1.008	[0.642–1.581]	0.947	0.980	[0.620–1.547]	0.930
Ethnicity						
Java	Ref.			Ref.		
Others	1.270	[0.679–2.377]	0.455	1.851	[0.974–3.517]	0.060
Contracted COVID-19						
Through public places	Ref.			Ref.		
Through COVID-19 carrier	1.099	[0.712–1.697]	0.669	0.939	[0.604–1.460]	0.780
Unknown	1.282	[0.827–1.897]	0.267	0.921	[0.590–1.436]	0.716
Place of Care						
Hospital	Ref.			Ref.		
Emergency hospital	0.886	[0.711–1.543]	0.551	1.023	[0.683–1.534]	0.912
Place of isolation						
At home or another place	Ref.			Ref.		
At hospital	1.048	[0.788–1.759]	0.813	0.891	[0.602–1.320]	0.566
Recovery duration						
Less than a month	Ref.			Ref.		
A month or more	0.790	[0.484–1.289]	0.346	0.816	[0.602–1.320]	0.566
Stigma						
Low	Ref.			Ref.		
Medium	4.874	[2.451–9.691]	0.000***	5.354	[2.671–10.734]	0.000***
High	1.744	[1.032–2.946]	0.038**	1.840	[1.058–3.199]	0.031**

*p<0.1

**p<0.05

***p<0.01; AOR: Adjusted Odds Ratio; CI: Confidence Interval

## Discussion

In the Indonesian setting, confirmed COVID-19 cases and deaths as well as recovered cases continue to be reported; however, research on issues related to stigma, QoL and mental health status among COVID-19 survivors is currently limited. COVID-19 survivors seem to be vulnerable in the community, putting them at the greatest risk in the general population. We found that a high level of stigma was positively correlated with lower QoL and mental health status among Indonesian COVID-19 survivors. Our findings point toward stigmatization among COVID-19 survivors and reveal the need to develop specific programs for targeted groups.

Pandemics may increase stigmatization, as previously observed during the severe acute respiratory syndrome (SARS) epidemic and the bubonic plague [28, 29]. The stigma and fear that has developed alongside COVID-19 is likely due to the uncertain characteristics and course of the disease as well as how it is treated. This is especially true when there are limited approved treatments with unpredictable outcomes, which may generate negative psychological responses. Thus, COVID-19 survivors are likely to be labeled and discriminated against because of the perceived connotations of and links to the disease [30]. In addition, most countries were not prepared for the pandemic, thus exacerbating chronic inequities and increasing the mortality rate [31–33]. A similar study from Hong Kong found that COVID-19 survivors experienced a high level of externalized stigmatization compared to those with HIV/AIDS and tuberculosis. The stigma that occurs in the community is due to the fear that survivors can still transmit COVID-19, which is due to a lack of accurate knowledge and information [34, 35] The stigma experienced by survivors can increase their suffering and cause them to hide symptoms to avoid discrimination. In addition, they may hide their medical history and information about disease transmission, which can facilitate transmission in the community and impact how the pandemic is controlled [30]. Thus, such an environment can fuel harmful stereotypes and undermine social cohesion. Moreover, stigma can lead people to physical violence and hate crimes [31]. Creating a safe environment and providing respectful care may result in better treatment for COVID-19 survivors when they return to their communities. In addition, governments should focus on programs for disseminating the facts about COVID-19 across sectors by community leaders, mass media, artists or social influencers [36]. The subsequent increase in knowledge can lessen the anxiety associated with the COVID-19 pandemic.

In our study, stigma among COVID-19 survivors remained a salient issue that was significantly associated with QoL. A high level of stigma was positively associated with lower QoL. These findings are consistent with those of studies conducted among health care workers in Italy and Egypt [37, 38]. Considering that stigma among COVID-19 survivors is a pressing issue for individuals, the community, and health care workers, there is still a lack of research into the relationship between stigma and QoL among COVID-19 survivors in the community. More data are needed to scrutinize the impact of stigma on individual QoL. The only study explaining the mechanism of stigma on QoL was conducted with regard to HIV [39]. In addition, our findings that COVID-19-related stigma has a significant correlation with survivors’ mental health are consistent with a previous study detailing how stigmatization is related to a high possibility of having poor mental health [40]. COVID-19 survivors may experience excess stress from stigma and discrimination, which may ultimately lead to mental disturbances. The stigmatization process occurs as a result of fear and being held responsible for contracting COVID-19 in the community. When this happens, individuals begin to gossip, become too interested in their COVID-19 experiences, and become wary of interacting with survivors. Such behavior leads to dread in disclosing a positive COVID-19 status as well as an unwillingness to meet new individuals, particularly those from high-risk groups [41]. Finally, negative effects on social interactions are associated with a reduction in the overall quality of life and mental health of the individual.

In this study, we found that females were less likely to experience stigma related to mental health than males; however, females present significantly higher levels of stress and anxiety and poorer mental health statuses [42]. Males also face problems associated with work, income, family, and life transition factors that have an impact on stress and mental disorders. Additionally, the lack of counseling facilities for men associated with stereotypes of masculinity needs further attention [43, 44]. During the COVID-19 pandemic, male survivors also experienced impacts on work, income, and self-actualization, which promote the development of mental disorders [45, 46]. COVID-19 survivors have struggled to lead a meaningful life and have been burdened with mental health issues [47, 48]. The significant correlation between men and mental health leading to worse outcomes in COVID-19 has been supported by other studies in China [49]. However, understanding and providing a psychological consultation room can help reduce the psychological burden experienced by males.

We also discovered that laborers and entrepreneurs were less likely to experience stigma related to mental health. Testing positive for COVID-19 while working as a laborer may cause insecurity due to lost work productivity. However, a good and conducive work environment can provide material, psychological and social support. Previous research has stated that a good work environment can provide support for COVID-19 survivors to recover quickly and promote enthusiasm during quarantine [50, 51]. Such support can be in the form of food, money, and daily necessities. The WHO stated that the effects of stigma on mental health among health care workers, patients, and survivors could be avoidable through adequate education through the media [6]. Understanding the primary drivers of misinformation is critical to preventing misjudgment in the community and increasing the sense of brotherhood among individuals.

In our research, the respondents were asked specific questions about several elements of their QoL and mental health. Standardized surveys measuring QoL and mental health status can reduce information bias [26], which we believe is a strength of our study. To our knowledge, this was the first analysis of stigma against Indonesian COVID-19 survivors associated with mental health indicators and QoL. The results call for urgent action to develop programs to destigmatize COVID-19 at every level, ranging from personal to policy. The main limitation of this study is its cross-sectional design, which cannot explain causality. Additionally, the study was conducted only in East Java Province; thus, the results of this study have limited generalizability because the respondents are representatives of the Indonesian population.

## Conclusions

A considerable proportion of the COVID-19 survivors in this cross-sectional study experienced COVID-19-related stigmatization at a medium level. Stigma among COVID-19 survivors has a close relationship with their QoL and mental health. These findings highlight the need for specific research and targeted interventions to address these issues for COVID-19 survivors. Given that Indonesia has suffered a high number of confirmed COVID-19 cases and deaths, the stigma experienced by COVID-19 survivors should be more broadly studied. COVID-19 survivors are a vulnerable group, and it is essential to identify new strategies to promote the well-being of this group as soon as possible. Our findings can inform policymakers to ensure the availability of a safe environment supported by respectful care. Urgent action is required to destigmatize COVID-19 at every level, ranging from personal to policy.
